# Serum 25(OH)D Level and Parathyroid Hormone in Chinese Adult Population: A Cross-Sectional Study in Guiyang Urban Community from Southeast of China

**DOI:** 10.1155/2013/150461

**Published:** 2013-08-28

**Authors:** Zhang Qiao, Shi Li-xing, Peng Nian-chun, Xu Shu-jing, Zhang Miao, Li Hong, Zhuang Hui-jun, Gong Ming-xian, Zhang Song, Wang Rui, Hu Ying, Zhang Jing-lu, Chen Shuang

**Affiliations:** Department of Endocrinology, The Hospital Affiliated to Guiyang Medical College, Guiyang 550004, China

## Abstract

*Objective*. To evaluate vitamin D status and serum parathyroid hormone (IPTH) of healthy adults living in Guiyang. *Design and Participants*. We conducted a cross-sectional evaluation in the General Community in Guiyang by cluster sampling method. The data was a part of 1510 participants (634 men, 876 women) aged 20–79 years median 45.2 years from November 2009 to February 2010 in Guiyang Health Measures Survey. *Measurements*. Aradioimmunoassay was used to measure the level of 25-hydroxyvitamin D [25(OH)D] and intact parathyroid hormone (iPTH). *Results*.The mean serum 25(OH)D level was (20.4 ± 9.0) ng/mL and the highest level among participants aged 40–59 years (22.8 ng/mL). The mean serum PTH level was (32.1 ± 13.7) pg/mL and the lowest level among participants aged 40–50 years (30.8 ng/mL). Serum 25(OH)D was below 50 nmol/liter in 52.3%, below 75 nmol/liter in 84.6%, and above 75 nmol/liter in 15.4% of the respondents. Secondary hyperparathyroidism was 5.4% (5.4% among men and 4.6% among women). The prevalence of secondary hyperparathyroidism increased (5.8%, 6.5%, and 7.1%, resp.) with decreasing serum 25(OH)D levels among subjects who were 30 to 20, 19.9 to 10, and <10 ng/mL, respectively. Serum 25(OH)D was inversely associated with serum PTH. *Conclusions*. Vitamin D insufficiency and its complication of secondary hyperparathyroidism are common.

## 1. Introduction 

Deficiency of Vitamin D is becoming a global public health problem [1]. Vitamin D plays important physiological roles in maintaining the balance of calcium and phosphorus metabolism and keeping normal bone mineral density levels. What is more, it acts as transcriptional factor in many cells [2, 3] Although the optimal concentration for over-all health is currently under debate, lower levels of vitamin D have been associated with a greater risk of osteomalacia in adult, increased risk of fracture, falls [4, 5], poor immunity, breast cancer, colorectal cancer and adenoma, cardiovascular diseases, and glucose metabolism disorders [6, 7]. Treating Vitamin D deficiency might bring extra benefit to many organs and systems beyond improving the bone and muscle health [8]. PTH is secreted in response to reduced calcium levels causing an increase in bone resorption and subsequently normalization of calcium levels. In case of vitamin D deficiency, secondary hyperparathyroidism resulting in increased bone turnover, increased bone loss, and “remodelling space” may subsequently increase fracture risk. Vitamin D status was affected by many factors such as geographical environment, physiological factors, and life style [1]. Data about vitamin D status in China were largely from north and eastern China. And very few studies were in large sample size by cluster sampling method. Here by using cluster sampling method, we evaluated vitamin D status and serum parathyroid hormone (IPTH) levels in 1510 healthy volunteers aged 20–79 yr (median age 45.2 years) and selected randomly from a community in Guiyang, a city in southwest of China, aiming to establish the epidemiology of the vitamin D status and secondary hyperparathyroidism in southwest China. 

## 2. Subjects and Methods

### 2.1. Subjects

The participants were recruited from a random selection of 1510 healthy volunteers, aged 20–79 yr (median age 45.2 years), resided in Guiyang over 10 years, and selected randomly from 7 districts in Guiyang by cluster sampling method. The number of Participants and gender ratio in all ages were determined according to the results of the fifth census in 2000. The participants were divided into three groups: youth (20–39 years), middle age (40–59 years), and senior (60–79 years) and received a questionnaire concerning medical history, medication, and lifestyle factors.

Participants were excluded for the following reasons: bedridden, dystrophia, chronic liver diseases, renal diseases, and parathyroid diseases. They were also excluded for refusing to give informed consent and having given birth within one year or currently lactating or under medication which might affect bone metabolism.

The questionnaire included basic information (age, gender, the history of fracture, and the age at menopause), educational level (≤6 years, 7–9 years, 10–12 years, and ≥13 years), smoking (daily smoking count × smoking years > 100 cigaret/years), drinking (drinking amount per day > 30 g), milk consumption (≥250 mL/day), outdoor activities, along with diabetes, hypertension, coronary heart disease, medication history, the supplement of vitamin D (≥250 IU/d), and calcium preparation (≥300 mg/day).

### 2.2. Anthropometric Measurements

Height was measured using a stadiometer to the nearest 0.1 m. Body weight was measured using a standard balance beam scale to the nearest 0.1 kg. BMI was calculated as body weight divided by the square of their height (kg/m^2^). Waist circumference was measured to the nearest 0.1 cm by applying the measuring tape horizontally midway between the lowest rib and the iliac crest after a normal expiration. Hip circumference was measured at the point yielding the maximum circumference over the greater trochanter, and waist/hip ratio (WHR) was calculated as waist divided by hip circumference. Participants in the present study were divided into 4 groups based on their BMI levels, including underweight (<18.5 kg/m^2^), normal (18.5–23.9 kg/m^2^), overweight (24–27.9 kg/m^2^), and obese (≥28 kg/m^2^), according to the guidelines for prevention and control of overweight and obesity in Chinese adults (2003). Waist circumference ≥85 cm and WHR ≥ 0.85 were abnormal for males, while waist circumference ≥80 cm and WHR ≥ 0.8 were abnormal for females. 

### 2.3. Biochemistry

Blood samples were obtained in the morning and immediately centrifuged and frozen. The serum samples were stored at −70 C. Serum 25(OH)D level and PTH were measured by automatica radioimmunoassay (Diasorin, Ltd, USA). The lower and upper detection limits of serum 25(OH)D level and PTH level are 1.65–59.65 ng/mL (1 ng/mL = 2.5 nmol/L), (0–2000 pg/mL) (1 pmol/L = 9.5 pg/mL), respectively. The interassay coefficients of variation (CV) of serum 25(OH)D and PTH were 8.8% and 2.4%, respectively. The intra-assay coefficients of variation (CV) of serum 25(OH)D and PTH were 11.1% and 4.9%, respectively. Serum calcium, phosphorus, and Cr were measured using automatic biochemical analyzer (Olympus AU5400). The analyses were carried out at the Endocrine Laboratory of the Guiyang Medical College Center. Quality control was maintained between investigators. The internal standard was used as control group when parallel determination was done in single sample. The interassay and the intra-assay variation coefficients of the kit was 4.9% and 2.4%, respectively. The normal reference range of iPTH level was 13–54 pg/m (1 pmol/L = 9.5 pg/mL). Also, serum calcium, phosphorus, and creatinine levels were measured on OL AU5400 automatic biochemical analyzer. 1510 subjects were measured anthropometric and tested of serum calcium, phosphorus, and creatinine levels. The serum 25(OH)D of 1494 subjects (627 males, 867 females) and the serum iPTH of 1417 subjects (588 males, 829 females) were tested.

The nutritional status of vitamin D was divided into 3 groups based on 25(OH)D levels [2]: vitamin D deficiency (25(OH)D < 20.0 ng/mL), vitamin D insufficiency (20.0 ng/mL ≤ 25(OH)D < 30.0 ng/mL) and vitamin D sufficiency (25(OH)D ≥ 30.0 ng/mL). Subjects with serum 25(OH)D < 30.0 ng/mL and the iPTH > 54 pg/mL were diagnosed with secondary hyperparathyroidism.

## 3. Statistical Analysis 

Epidata 2.00 was used for the basic data entry. Data are expressed as means ± SD or percentage. Continuous or categorical data were analyzed utilizing ANOVA or *χ* ^2^ tests. Spearman correlation coefficients were calculated to examine the association between 25(OH)D levels and measures of variables. Determinants of vitamin D sufficiency were evaluated using logistic regression analysis including vitamin D status as a dichotomous dependent variable and age, calcium supplements, smoking, alcohol, sports, WHR, and BMI as independent variables. Multiple regression analyses were performed with 25(OH)D as the dependent variable. All statistics were performed with SPSS 11.0. *P* values < 0.05 were considered significant. 

## 4. Results

### 4.1. Study Subjects Characteristics

Median age of the participants was 45.2 (range: 20–79) years, and 58% were women. There were no gender differences in average 25-OH D. The average 25(OH)D level was 20.4 ng/mL, close to represent range considered to represent vitamin D deficiency (25(OH)D < 20 ng/mL). However, the average PTH level was within the normal range. On average, men had greater BMI, waist circumference, WHR, serum Creatinine, and PTH than women (all *P* < 0.01). The average serum Phosphate was greater in women than men (*P* = 0.03). The average total calcium and physical activity did not differ between men and women. Men had higher percentage of alcohol and smoking but lower percentage of milk intakes, calcium, and vitamin D supplementation than women (Table 1).

The mean serum 25(OH)D level was the highest among participants aged from 40 to 59 (22.8 ng/mL) (all *P* < 0.01). However, the mean serum PTH level was the lowest among participants aged from 40 to 59 (30.8 ng/mL). On average, men of middle age and senior had higher serum PTH and serum Phosphate than women (all *P* < 0.01). Moreover, old age men of had higher serum 25(OH)D than women (*P* = 0.005). Serum 25(OH)D was below 50 nmol/liter in 52.3%, below 75 nmol/liter in 84.6%, and above 75 nmol/liter in 15.4% of the respondents (Table 2).

The prevalence of secondary hyperparathyroidism was 5.4% (5.4% among men and 4.6% among women). There was no statistical difference between the two genders (*P* = 0.29). Moreover, the prevalence of secondary hyperparathyroidism was 5.2% and 3.7% among premenopausal and postmenopausal women, respectively. The prevalence of secondary hyperparathyroidism increased (5.8% (27), 6.5% (37), and 7.1% (12), *P* = 0.001) with decreasing serum 25(OH)D levels among subjects whose levels were (30–20) ng/mL, (29.9–10) ng/mL, and <10 ng/mL, respectively.

### 4.2. Relationships of 25-Hydroxyvitamin D and Parathyroid Hormone Levels with Anthropometric Measures BMI, Waist Circumference, and WHR

In different BMI groups, serum 25(OH)D levels trended higher when BMI progressed from underweight group to obese groups between males and females (all *P* < 0.05). Comparing with the underweight group, the serum 25(OH)D levels in normal, overweight, and obese groups were higher (all *P* < 0.01). However, there was no statistical difference of the serum 25(OH)D levels among the normal, overweight, and obese groups. There was no significant difference between subjects of normal and abnormal waist circumference. The serum 25(OH)D level in normal WHR females was higher than that of abnormal WHR ones (*P* = 0.043). However, the difference did not exist among the males (Table 3).

### 4.3. Educational Level

The serum 25(OH)D concentrations were statistically different among the subjects (both males and females) with different educational background, (all *P* < 0.001), (Table 3). The lowest serum 25(OH)D concentrations were detected in the subjects with ≥13 years of education. 74.2% of subjects with ≥13 years of education were vitamin D deficiency.

### 4.4. Living Habits

Compared with the control, serum 25(OH)D levels were higher in the higher daily vitamin D, calcium, milk intakes, non-smoking and, longer times outdoor activity groups (≥60 min/day), although it was only significantly different for smoking, drinking milk, and calcium supplementation in the women (all *P* < 0.05), whether the subjects with alcohol intake were not statistically significant (Table 3). Similar result was found in subjects with calcium supplement and vitamin D, while the statistical difference only existed among the females (*P* = 0.009, Table 3).

### 4.5. Relationships of 25(OH)D and PTH Levels with Anthropometric Measures, Physical Activity, Supplements of Calcium and Vitamin D, Smoking, and Drinking Milk

The univariate analysis showed that the following factors: age, outdoor activities, smoking, and WHR were associated with 25(OH)D levels (*P* < 0.05). Those factors were then analyzed by the multiple logistic regression and there was still relevance. The risks of abnormal vitamin D levels in youth and senior groups were higher than the middle age group, with odds ratio (OR) as 1.846, 1.918 (*P* < 0.001, *P* < 0.003, resp.). Comparing with the control group, higher risk of vitamin D deficiency was associated with WHR abnormality, less outdoor activities (<60 min/day), and smoking (OR = 1.559, 1.490, 1.505, *P* = 0.006, 0.048, 0.049, resp., Tables 3 and 4). 

Serum iPTH status was inversely associated with serum 25(OH)D level while positively associated with gender, serum total calcium, serum creatinine, waist circumference, WHR, height, and weight (all *P* < 0.05) (Table 5).

## 5. Discussions

Most studies demonstrated that serum 25(OH)D was associated with serum PTH. Although the optimal concentration for overall health is currently under debate, the cut-off value of serum 25(OH)D level was determined at the level which was low enough to inhibit the elevated of PTH. At present, the well-recognized cut-off value for vitamin D deficiency was 30 ng/mL [2, 9–11].

The status of vitamin D was affected by many factors such as geographical location, physiological factors, and life style [1]. Serum 25(OH)D concentration of human in winter was lower than that in summer. Therefore, people living far away from the equator tend to have lower vitamin D levels than those who live nearer [12]. However, Previous studies on the postmenopausal women and other population showed that deficiency of vitamin D (25(OH)D < 30 ng/m) commonly exists among the south and southeast Asia such as Thailand, Malaysia, Japan, and Korea (47%, 49%, 90%, and 92%, resp.) [13]. Most studies on youth females and other population have demonstrated that the prevalence of deficiency of vitamin D were from 50% to 90% from Beijing, Hong Kong [14], Shanghai [15], and Shenyang [16].

Guizhou province is located in southwest of China with relatively fewer sunshine comparing to other parts of the country. Guiyang is located at 26.5 north latitude (26.5°N), the average cloudy days were 235.1 days, and the annually mean hours of sunshine were 1148.3 h. Our study showed that the average serum 25(OH)D level of 20.4 ng/mL, is nearly higher limit considered to represent vitamin D deficiency (25(OH)D < 20 ng/mL) in winter and spring. Serum 25(OH)D was below 50 nmol/liter in 52.3% and below 75 nmol/liter in 84.6%. This may be associated with the season when this survey was conducted, less outdoor activities (took up 20%) of the subjects, and less vitamin D supplement (only took up 3%). 

Vitamin D status was also related to age and gender [1, 2]. A study in Canada [17] found that vitamin D status of children (aged 6–11) was the best because of the large milk drinking quantity, then followed by the 60−79 age group because of the intake of the vitamin D supplements, with the worst among the 20–30 youth age group. While in our study, the result showed that serum 25(OH)D concentration of the 40–59 age group was higher than that of the 20–39 and 60–79 age groups, among which the 20–39 age group was the lowest, which was similar to that result. The possible reasons were as follows: (1) most of the youth were working indoor. As a result, they have less time spent in the outside hence less sun exposure. In addition, they did not have the habit of drinking milk; (2) the ability of synthesizing and absorbing vitamin D was decreased in the seniors [17]. Besides that, a study from the Netherlands by Kuchuk et al. [11] found that among the 1319 volunteers (aged 65–88), serum 25(OH)D concentration of males was higher than that of the females which was the same as our result. It may be the reason that the males in senior group did more outdoor activities and ate more varieties of foods than females. It was indicated that serum iPTH status was associated with gender, age, and serum creatinine [18]. Moreover, a negative correlation existed between the serum iPTH status and 25(OH)D concentration [18], which was similar to our results.

Currently, the relationship of body fat index with 25(OH)D and serum iPTH levels remains controversial [18–21]. Most of the reports showed that the 25(OH)D concentration was negatively correlated with serum iPTH status and also with waist circumstance, BMI, hip circumstance, and total body fat [18, 22]. In our present work, it was showed that the serum 25(OH)D concentration was associated with WHR and negatively associated with serum iPTH, while serum iPTH was associated with weight, waist circumstance, and WHR. Lu et al. found no statistical difference of the serum 25(OH)D concentrations between subjects with BMI ≥ 24 Kg/m^2^ and BMI< 24 Kg/m^2^ in Chinese middle-aged and senior citizens, which was similar with our results but not completely consistent to the results of western studies. The difference might relate to the difference of reference value of BMI. In our study, low serum 25(OH)D concentration was found to be associated with the resulting elevated iPTH, waist circumstance, and/or WHR, which suggested that abdominal obesity might be the risk factor of adult vitamin D deficiency and secondary hyperparathyroidism in Guiyang.

In our work, we found that subjects with different education backgrounds had different serum 25(OH)D concentration and vitamin D status, which was the same with a previous study [15]. Besides that, smoking was identified as a risk factor of the vitamin D deficiency. The smokers may get 1.5 times higher risk to develop vitamin D deficiency than nonsmokers. The serum 25(OH)D concentration of the smokers was obviously lower than that of nonsmokers, which was the same as a previous study[19], while its mechanism remained unclear. In addition, we found that the serum 25(OH)D concentrations in subjects who drank milk and had more outdoor activities were higher [2, 21], but no correlations were found. It might be the reason that the quantities of milk drinking, the intake of vitamin D, and calcium supplements among our subjects were less. 

Low level of 25(OH)D might increase serum iPTH which might cause SHPT. In our study, we found that 5.4% of the subjects with abnormal vitamin D status developed SHPT which was similar to the data from Hong Kong (6.3%) [23]. We found that the prevalence of SHPT increased with the decrease of the serum 25(OH)D level. Low concentration of serum 25(OH)D and the resulting elevated iPTH were associated with waist circumstence and WHR. These results indicated that correcting vitamin D deficiency and its complication of SHPT might benefit not only bone health but could also probably prevent many obesity-related diseases, including metabolic syndrome, diabetes, and hypertension. 

Our cross-sectional study in healthy adult in a city in southwest of China demonstrated that decreased 25(OH)D and raised PTH levels were both linked to anthropometric measures and living habits. We reported observations in heath adults cohort linking 25(OH)D closely to age, outdoors activity, WHR, and smoking and linking PTH closely to 25(OH)D level, gender, serum total calcium, serum creatinine, waist circumference, WHR, height, and weight.

This study is the first epidemiological survey for vitamin D status and secondary hyperparathyroidism in southwest China. The study was performed by stratified cluster sampling method with a large sample size. The study subjects were ranged from 20 to 79 years old. In this study, we analyzed the factors that might be associated with the vitamin D status and iPTH levels in a wide range such as age, gender, weight, BMI, waist circumstence, WHR, smoking, drinking, educational levels, outdoor activities, milk drinking, serum calcium, phosphorus, and creatinine. The result has good representativeness. However, there was still some limits in our work. For example, the investing season was only winter and spring and the style of sports and the qualities of the milk drinking were not recorded, which might to some extent affect the results. 

## 6. Conclusion

Vitamin D insufficiency and its complication of secondary hyperparathyroidism is common in Guiyang. 

## Figures and Tables

**Table 1:** Baseline characteristics (*n* = 1510).

Parameter	Total sample *n* (%)/$\overset{-}{x}\pm s$	Men *n* (%)/$\overset{-}{x}\pm s$	Women *n* (%)/$\overset{-}{x}\pm s$
Age (yr)			
20–	267 (17.7)	115 (18.1)	152 (17.4)
30–	351 (23.2)	148 (23.3)	203 (23.2)
40–	329 (21.8)	135 (21.3)	194 (22.1)
50–	284 (18.8)	118 (18.6)	166 (18.9)
60–	155 (10.3)	60 (9.5)	95 (10.8)
70–	124 (8.2)	58 (9.1)	66 (7.5)
20–79	1510 (100)	634 (42.0)	876 (58.0)
Alcohol (%)	444 (29.4)	376 (59.3)	68 (7.8)^a^
Smoker (%)	250 (16.6)	206 (32.5)	44 (5.0)^a^
Drinking milk	297 (19.7)	118 (18.6)	179 (20.4)
Calcium supplementation	179 (11.9)	51 (8.0)	128 (14.6)
Vitamin D supplementation	48 (3.2)	15 (2.4)	33 (3.8)
Outdoors activity			
≤30 min/day	842 (55.8)	377 (59.5)	465 (53.1)
30–60 min/day	351 (23.2)	146 (23.0)	205 (23.4)
≥60 min/day	314 (20.8)	109 (17.2)	205 (23.4)
Education (yr)			
0–6	205 (13.6)	51 (8.0)	154 (17.6)
7–9	408 (27.0)	144 (22.7)	264 (30.1)
10–12	353 (23.4)	142 (22.4)	211 (24.1)
≥13	544 (36.0)	297 (46.8)	247 (28.2)
BMI (Kg/m^2^)	23.7 ± 3.6	24.1 ± 3.3	23.4 ± 3.7^a^
Waist circumference (cm)	81.6 ± 9.8	85.4 ± 8.9	78.7 ± 9.4^a^
Waist/hip ratio	0.86 ± 0.06	0.89 ± 0.06	0.83 ± 0.06^a^
Total calcium (mmol/L)	2.4 ± 0.2	2.4 ± 0.2	2.4 ± 0.2
Phosphate (mmol/L)	1.1 ± 0.2	1.0 ± 0.2	1.1 ± 0.1^a^
Creatinine (umol/L)	63.7 ± 14.7	74.6 ± 12.7	55.8 ± 10.2^a^
25(OH)D (ng/mL)	20.4 ± 9.0	20.8 ± 9.7	20.1 ± 8.5
iPTH (pg/mL)	32.1 ± 13.7	33.5 ± 12.4	30.5 ± 13.6^a^

Values are presented as mean ± SD. For conversion of 25(OH)D from nmol/liter to ng/mL, divide by 2.496; for conversion of PTH from pmol/liter to pg/mL, multiply by 11.1. Distribution of PTH and of a number of chronic diseases was skewed. Median (interquartile range) is presented; serum 25(OH)D was assessed in a sample (*n* = 1494) and iPTH (*n* = 1414). ^a^ *P* < 0.01 as compared to men.

**Table 2:** Differences in the mean values of BMI, Serum 25(OH)D, PTH, calcium, Phosphorus, and Creatinine in different age groups (*n* = 1510).

Parameter	Youth (*n* = 618)			Middle age (*n* = 613)			Senior (*n* = 279)		
	Total sample	Men	Women	Total sample	Men	Women	Total sample	Men	Women
Age (yr)	30.8 ± 5.7	30.4 ± 5.5	31.1 ± 5.8	49.2 ± 5.5	49.2 ± 5.8	49.2 ± 5.8	68.1 ± 4.5	68.9 ± 4.2	67.5 ± 4.6
BMI (kg/m^2^)	22.5 ± 3.6	23.5 ± 3.6	21.7 ± 3.4	24.3 ± 3.3	24.5 ± 3.1	24.2 ± 3.4	25.0 ± 3.4	24.6 ± 2.9	25.4 ± 3.6
25(OH)D (ng/mL)	18.2 ± 9.2^a^	17.6 ± 9.5^a^	18.7 ± 9.0	22.8 ± 8.7	23.4 ± 8.5	22.4 ± 8.1	19.9 ± 7.8^a^	22.2 ± 8.4	18.2 ± 6.9^b^
iPTH (pg/mL)	33.0 ± 13.2^a^	32.3 ± 12.3	33.5 ± 13.8	30.8 ± 13.3	35.0 ± 13.9	27.9 ± 12.1^b^	32.8 ± 15.2^a^	35.6 ± 14.1	30.8 ± 15.7^b^
Total calcium (mmol/mL)	2.4 ± 0.1	2.4 ± 0.1	2.4 ± 0.1	2.4 ± 0.2	2.4 ± 0.1	2.4 ± 0.2	2.4 ± 0.2	2.4 ± 0.2	2.4 ± 0.2
Phosphate (mmol/mL)	1.1 ± 0.2	1.1 ± 0.2	1.1 ± 0.1	1.1 ± 0.2	1.0 ± 0.2	1.1 ± 0.1^b^	1.0 ± 0.2	1.0 ± 0.1	1.1 ± 0.1^b^
Creatinine (umol/L)	67.1 ± 14.1	69.3 ± 11.5	66.5 ± 14.7	73.8 ± 10.4	74.2 ± 12.3	71.9 ± 11.1	76.6 ± 13.1^c^	78.4 ± 11.5	74.7 ± 10.6

Values are presented as mean ± SD. For conversion of 25(OH)D from nmol/liter to ng/mL, divide by 2.496; for conversion of PTH from pmol/liter to pg/mL, multiply by 11.1. Distribution of PTH was skewed. Median (interquartile range) are presented; serum 25(OH)D was assessed in a sample (*n* = 1494) and iPTH (*n* = 1414). ^a^ *P* < 0.01, as compared to the middle age category, ^b^ *P* < 0.01, as compared to men, ^c^ *P* < 0.01, as compared to youth category.

**Table 3:** 25(OH)D concentrations by participant characteristics (*n* = 1510).

Parameter	25(OH)D concentrations			
	Male		Female	
BMI (Kg/m^2^)		0.021		0.004
<18.5	17.6 ± 10.8		16.7 ± 10.0	
18.5–23.9	19.9 ± 10.2		20.7 ± 8.7	
24–27.9	22.1 ± 9.2		20.1 ± 7.8	
≥28	20.1 ± 8.6		19.5 ± 7.6	
Waist circumference		0.45		0.78
Normal	20.3 ± 10.2		20.2 ± 8.9	
Abnormal	21.2 ± 9.2		20.1 ± 7.9	
WHR		0.075		0.043
Normal	20.0 ± 10.7		21.0 ± 9.0	
Abnormal	20.9 ± 9.4		19.7 ± 8.3	
Education (yr)		<0.001		<0.001
0–6	25.6 ± 7.6		20.1 ± 7.6	
7–9	22.7 ± 10.2		21.0 ± 7.8	
10–12	20.7 ± 9.0		22.0 ± 8.6	
≥13	19.0 ± 9.6		17.6 ± 9.0	
Outdoors activity		0.007		0.005
<30 min/day	20.2 ± 9.7		19.9 ± 8.7	
30–60 min/day	19.9 ± 9.3		19.1 ± 7.4	
≥60 min/day	23.4 ± 9.0		21.7 ± 8.7	
Smoking		0.006		0.009
Yes	18.3 ± 9.6		17.8 ± 8.9	
No	21.4 ± 9.7		20.0 ± 8.4	
Drinking alcohol		0.064		0.078
Yes	21.3 ± 9.4		20.8 ± 8.9	
No	22.4 ± 9.4		20.4 ± 8.4	
Drinking milk		0.017		0.042
Yes	22.8 ± 10.1		21.2 ± 8.2	
No	20.3 ± 9.5		18.6 ± 8.5	
Calcium supplementation		0.060		0.009
Yes	23.1 ± 9.8		21.9 ± 8.5	
No	20.5 ± 9.7		19.8 ± 8.4	
Vitamin D supplementation		0.059		0.054
Yes	23.2 ± 9.5		21.9 ± 7.2	
No	20.7 ± 9.7		20.0 ± 8.5	

Data are expressed as means ± SD, the comparison between each group was tested by ANOVA or chi-square.

**Table 4:** Multiple factor logistic regression of low serum 25(OH)D of less than 30 ng/mL.

Variables	*β* (SE)	OR (95% CI)	*P* value
Age			
20–39 yr	0.613 (0.167)	1.846 (1.332–2.559)	<0.001
40–59 yr	—	—	
60–79 yr	0.651 (0.216)	1.918 (1.256–2.929)	0.003
Waist/hip ratio			
Normal			
Abnormal	0.444 (0.161)	1.559 (1.137–2.138)	0.006
Outdoors activity			
<30 min/day	0.290 (0.179)	1.337 (0.941–1.898)	0.105
30–60 min/day	0.401 (0.172)	1.490 (0.970–2.228)	0.050
≥60 min/day	—	—	
Smoking			
Yes	0.435 (0.211)	1.506 (0.097–2.337)	0.049
No	—	—	

**Table 5:** Multiple factor logistic regression of high serum PTH of greater than normal levels.

Variables	*β* coefficient	*P* value
Sex	−0.114	<0.001
25(OH)D	−0.101	<0.001
Total calcium (mmol/mL)	−0.101	0.002
Creatinine (umol/L)	0.061	0.022
Height	0.108	<0.001
Weight	0.072	0.007
Waist/hip ratio	0.079	0.003
Waist circumference (cm)	0.082	0.01
